# The Surgical Aspect of Gastric Ulcer1Read before the Section on Medicine, Buffalo Academy of Medicine, January 15, 1907.

**Published:** 1907-03

**Authors:** Eugene A. Smith

**Affiliations:** Buffalo, N. Y.; 1018 Main Street


### BUFFALO MEDICAL JOURNAL.

VOL. LXII. MARCH 1907. No. 8

### ORIGINAL COMMUNICATIONS.

### The Surgical Aspect of Gastric Ulcer.[1]

BY EUGENE A. SMITH, M. D., Buffalo, N. Y.

TO my mind a discussion of the surgical aspect of gastric ulcer in no way indicates a conflict between the surgical and medical viewpoints of the subject, nor do I mean to say that the surgical or operative treatment of gastric ulcer is of paramount importance in the treatment of all such cases. I am free to say this subject has a broad medical basis of diagnosis and treatment upon which in appropriate cases treatment rests. The burden of diagnosis falls upon the internist in fact, and also the treatment in practically all cases for the first few days, weeks or months, sometimes even for years. I think it is fair to say that the importance of the discussion of the medical aspects of gastric ulcer is not lessened, but the essential features of diagnosis and treatment are emphasized when it is granted that certain classes of gastric ulcer need surgical treatment.

In a free discussion leading to the classification of cases which are surgical, I may go over ground already ably covered by Dr. Woehnert in his discussion of the etiology, symptomatology and treatment of gastric ulcer. This risk of repetition I must run, or feel that as a surgeon I am reduced to the position of a mere mechanic for whom diagnosis is made and a command issued by the internist to do for one case a gastrojejunostomy, for another a pyloroplasty, pylorectomy, a gastrostomy, a gastroplication or a gastrectomy. If the internist and surgeon do not work in harmony in these cases, we find the former timid and procrastinating, the latter making too many exploratory operations, or adding to the records of needless operations. Under such circumstances both internist and surgeon are prone to make false deductions from their experiences, and progress in scientific medicine is retarded.

---

1. Read before the Section on Medicine, Buffalo Academy of Medicine, January 15, 1907.

It is said that from five to thirteen per cent. of people suffer at some time of their lives from gastric ulcer, and in the different authorities, statistics can be quoted of interest from many points of view as to age of patient, location of ulcers, number of ulcers, single or multiple, number in which hemorrhage is a feature, proportion of cases in which perforation or malignancy later develops, and so on. Upon these phases of the subject it is not my purpose to dwell.

Diagnosis of gastric ulcer is not possible in all cases. Hemmeter said at the 1906 meeting of the American Medical Association that diagnosis of gastric ulcer depends largely upon the exercise of the sixth sense. The usual procedure in arriving at diagnosis is by exclusion of other diseases. In many cases of acute or chronic dyspepsia (using the word dyspepsia in a broad sense, including patients suffering from indigestion) regulation laboratory methods, examination of stomach contents and stools, and a close study of the history in the individual case help us with reasonable certainty in the diagnosis of gastric ulcer.

Diagnosis being made, we find prognosis most uncertain. The acute dyspeptic of this week may be a patient with perforating ulcer or acute hemorrhage next week. The acute or chronic dyspeptic of this month may be entirely relieved under proper medical treatment in another month. When the dyspeptic is brought to operation on a diagnosis of ulcer of chronic type, internist and surgeon must realize that there is always an exploratory feature in an operation in suspected cases of gastric ulcer. In my own experience in consultation work, as a result of close study or as a result of exploratory operation, some supposed cases of gastric ulcer were found to be cases of chronic cholecystitis with calculi, some were cases of reflex gastric neurosis due to nephroptosia, chronic appendicitis, chronic pelvic disease, or beginning malignancy. One case diagnosticated as possible gastric ulcer was found to be a massive hydrothorax of the right side with marked downward displacement of the liver.

Hemmeter is working at present to develop a pathognomonic sign of gastric ulcer by *x*-ray and fluoroscopic findings upon giving bismuth to patients suffering from gastric ulcer. He says there seems to be a peculiar affinity between the exudate in these ulcers and bismuth, the *x*-ray plainly showing it. It seems to me, however, that in cancer with erosion the same findings would be present.

I had the pleasure of watching Moynihan's work in Leeds for two days last June. In two cases out of four he demonstrated a sign of gastric ulcer the day before he operated upon them. Gently pinching up small areas of skin all over the epigastrium and adjoining regions between the first finger and thumb he asks the patients to say when the pinching hurts. The pinching is not severe enough to cause pain, excepting over the site of ulcer, which it therefore indicates. Moynihan is not able to give a satisfactory explanation for the occurrence of pain over the site of ulcer elicited in this way, but he offers as a theory to explain the phenomenon, the possibility of a reflex existing between the stomach and the overlying skin area, so that a corresponding skin area of sensitiveness is found over an ulcerated area in the viscus below. On the following day after a clinical discussion of these four cases in the wards of the Leeds General Hospital, Moynihan verified his diagnosis of the site of ulcer in making gastrojejunostomies. In the two cases in which he failed positively to elicit the sign, the house surgeon had previously on the same day developed the sign, and ulcer was found as he predicted. Moynihan's failure to bring out the sign he ascribed to tiring out the reflex, which he finds can be done readily if repeated several times within one day. To determine the obstructive results of gastric ulcer Moynihan makes free use of Seidlitz powder inflation, excepting in cases that have had recent hemorrhages.

From the clinical point of view the surgical treatment of gastric ulcer must be based on a clinical classification of gastric ulcers. The fine distinctions which the pathologist is able to make between the different types of ulcers are not possible at the bedside. It follows, therefore, that when the patient is brought to the operating table, the surgical operation planned for the condition diagnosticated to be present may not be the procedure indicated, and another quite different operation may be demanded by the condition found. In several of my cases the presence of cancer, for instance, has had this effect.

The so-called peptic ulcer, the gastric ulcer beginning to undergo malignant degeneration, traumatic ulcer, the large and small erosions, and the villous or hemorrhagic areas of the pathologist present symptoms so much alike that clinical differential diagnosis can not be made between them. Clinically we base a classification of gastric ulcers on the duration of the disease and the severity of the clinical symptoms, dividing them into acute and chronic ulcers.

The acute ulcer, excepting in the complications of perforation or hemorrhage, belongs to the domain of the internist. The dividing line between acute and chronic ulcers is difficult to draw. The internist who is not liberal enough to consider surgery as a means of relief or often a cure in the treatment of gastric ulcer will lose patients that he ought to save in the long run of his cases. The surgeon who is ready to operate on all cases presenting some symptoms of gastric ulcer,—for instance hematamesis or melena,—will resort to unnecessary operations and may turn the scale against a patient in certain cases where operation is not indicated.

Chronic ulcer, by which we usually mean to imply the indurated or calloused ulcer due to a process several weeks or months in duration, and as a rule accompanied by the symptoms of chronic dyspepsia, should be transferred by the internist to the surgeon for operative treatment. The indications for operative treatment in this class of ulcers is positive when perforation of the stomach occurs, when hemorrhages are recurrent and large, and when signs and symptoms of obstruction to the gastric outflow develop. Relapses after improvement or supposed cure constitute a cause for operation. Invalidism due to gastric ulcer and continued underfeeding call for operation because of the lowered vitality which ensues and which invites, in a large percentage of cases, complications that end fatally.

While operative treatment is indicated for frequent and debilitating hemorrhages and also for repetition of one severe hemorrhage, a patient suffering from a single severe hemorrhage is best treated medically. I cite the following history in support of this latter statement, adding as a note of explanation that operative interference at the time I saw the patient would probably have resulted in his death by adding the shock of surgical operation to the threatening collapse of serious hemorrhage. I do not mean to say that a later operation for drainage of the stomach was not indicated; in fact, I so advised.

On April 24, 1905, Dr. Hitzel asked me to see Henry G., a married man 57 years old, who had been a heavy drinker and a large eater. For years he had belching and water brash. In the afternoon of the day I saw him he ate heartily of roast beef and after a long drive during which he was chilled, drank a hot sling. Shortly afterward he vomited a large quantity of blood, and again at 6:30 and at 8 o'clock he vomited blood, one to two pints each time. I saw him about nine in the evening. On examination I found a corpulent man with moderate arteriosclerosis, tender epigastrium; pulse of 120 and weak; temperature 99½. He was blanched. Deciding against operation, he was put upon rectal alimentation, ice and adrenalin internally with morphine subcutaneously. He had no more hemorrhages. A year later, on March 22, 1906, he came to consult me. He said he had no vomiting, no loss of flesh, but had the same symptoms of belching and water brash and dyspepsia. I found pronounced epigastric tenderness and gastric splashing on succussion an hour after his meal. He declined operation. Dr. Hitzel reports that he became worse and entered the General Hospital in October and died there October 20, 1906. Post mortem was not obtained, but he died of general peritonitis a few days after the onset of typical symptoms of perforation of gastric ulcer. He refused operation, sinking to his death in the fancied security which the morphine he demanded gave him in the relief from pain.

In my own practice the summons to see patients suffering from hematemesis due to gastric ulcer has come as a rule when the patient was so exsanguinated that operation was contraindicated. In fact, some of them were moribund. Several histories of patients in this class might be cited. When operation is undertaken because of recurring hemorrhages, and the ulcer is found after opening the stomach, excision of the ulcer or purse string suture of the area should be made, after which the peritoneal surfaces should be apposed by seromuscular sutures. Following this, with patients in good condition, if the ulcer is so situated that later cicatricial contraction will interfere with gastric mobility and drainage, a gastrojejunostomy should be done. If hemorrhages have not been serious in nature, and the operation is done for ulcer of chronic type, direct attack upon the ulcer to check hemorrhage is not necessary; gastrojejunostomy will suffice according to Moynihan. By drainage, distention of the stomach is overcome and hemorrhage thereupon ceases he argues. Perforation of gastric ulcer may occur in acute ulcer, but is more apt to occur in chronic ulcers.

All authorities are agreed that perforation is an indicacation for immediate operation. Statistics prove that prognosis, if operation is done within six hours of perforation, is good, but after ten hours the majority of patients die. Moynihan reports twenty-two cases of acute perforation operated upon with eight deaths. Mayo reports seven acute perforations operated upon with two deaths. The disastrous result of acute perforation shows itself first as a local peritonitis, rapidly spreading to become a general suppurative peritonitis. The few cases of perforation in which the local peritonitis is limited by adhesions, or ends in localised abscess, are the exceptions which prove the rule that perforations cause acute general peritonitis and are fatal. When cases of chronic dyspepsia develop sudden onset of violent episgastric pain, quickly diffusing and becoming general abdominal pain, rapidly increasing pulse, frequent respirations and the facial expression of shock with or without vomiting, whether previously treated for gastric ulcer or not, the attending physician should diagnosticate the occurrence of this most serious accident. A case of this type came to me for operation with a diagnosis of acute perforative appendicitis. In these cases sutures of the perforation longitudinally in the wall of the stomach, or transversely if in the region of the pylorus, with pelvic drainage if general peritonitis has developed, followed by Murphy's continuous salt solution per rectum, and propping the patient in the Fowler's half sitting posture, give the best results. A typical case of this kind came under my observation quite recently, December 17, 1906, in consultation with Dr. Hayd.

Mr. D., 41 years old, a mail clerk, had hematemesis thirteen years ago, and since that time has been a dyspeptic and was treated by Dr. Hayd for dilatation of the stomach by lavage and dieting during last summer. At times in washing out the stomach, vegetables eaten two and three days before the treatment were found in the stomach contents. He declined operative treatment. He improved under the medical treatment, but was constantly on diet, sallow, anemic and emaciated. At one o'clock in the morning of December 17, he was taken with violent abdominal pain without vomiting. A physician was called who gave him a quarter of a grain of morphine hypodermically every hour for three doses. Dr. Hayd saw him at half past eight in the morning and I saw him at nine o'clock. His pulse was 92, small and weak, respiration 36, temperature 97, and taken per rectum 99. He was cyanosed. The abdomen was rigid and tender generally, but most exquisitely tender in the epigastrium and just below the free border of the ribs over the pylorus. The stools did not contain blood. Agreeing in Dr. Hayd's diagnosis of perforating ulcer of the stomach we finally obtained the patient's consent for an operation. I assisted in the operation, which was done at the Deaconness's Hospital at eleven o'clock. Perforating ulcer was found on the upper surface of the lesser curvature of the stomach near the pyloric end. There was dense infiltration and thickening all about the small opening which just admitted a probe. Through this opening fluid material and gas welled from the stomach. A general peritonitis was fully developed and the pelvis was later found full of seropurulent and malodorous fluid. Closure of the ulcer was made by sewing stomach folds longitudinally over it. A posterior gastrojejunostomy was next done. The abdominal wound was then closed. A stab wound of the hypogastrium was made and pelvic drainage established. The patient did well, taking nourishment freely after the fourth day and gained steadily until the ninth day, when evidences of pneumonia were detected. The pneumonic process rapidly increased, and he died on the 11th day.

Exploratory operations are to be recommended in obscure cases presenting gastric dyspeptic symptoms and in which gastric ulcer is suspected on some occasions, and I have previously mentioned in this paper some of the findings. The following two cases I report not because they are cases of gastric ulcer, but because ulcer or a sequela was suspected, and differential diagnosis could not be made.

On April 23, 1900, I saw Mrs. D. with Dr. Wetzel. She said she was sixty-four years old, a widow who had borne several children. Her health had always been good until a few months before I saw her, when she began having indigestion. At first she treated herself, and then going to a physician was treated for dyspepsia. Her trouble increased, vomiting began and constipation became more and more pronounced. She took food freely, drinking milk in large quantities, and lost but little in weight. Taking food a part of the day, she would vomit the rest of the day. For a few days before I saw her the vomited material was "bad smelling" as she expressed it, and at times was large in quantity, nearly filling a wash basin. While I was present she vomited, and I was able to verify her own description of the amount of fluid vomited and its odor. Her stools were dark and made up of marble-sized lumps.

On examination I found the patient in general good condition without evidence of abdominal tumor, but with pronounced gastric dilatation and splashing on succussion. I operated on April 27, finding a distended, dilated stomach with thin walls and prominent, distended veins. The small intestine from the duodenum down was completely empty and no bigger than a lead pencil. The colon was empty and small. From the pylorus downward a firm dense mass bound down the duodenum and posterior surface of the stomach. I therefore made an anterior gastroenterostomy, using a Murphy button. Operation was readily acomplished, the only difficulty being the control of the fluid which tended to well from the overfull stomach. She recovered quickly after operation and did well for four months, digesting food without trouble. She had one source of worry in which I shared; the Murphy button did not come away. In early September, she began having dyspeptic symptoms again. She lost flesh and in December developed jaundice. A vague tumor could be made out between the navel and free border of ribs at this time. She died January 18, 1901. On post mortem I found cancer involving pylorus and duodenum with metastasis in the liver. The Murphy button had passed from the stomach into the duodenum and was found about three inches from the pyloric end of the stomach where it was stopped by complete occlusion of the duodenum. It had done no harm in this position.

The anterior loop operation is to be regarded as an operation of necessity, when gastroenterostomy is to be performed. It is used when the posterior surface of the stomach cannot be reached.

The following interesting history of another obscure case is reported, diagnosis being made by an exploratory incision:

October 25, 1896, I operated on S. H. at the German Deaconness's Hospital. He gave the following history: He was a married man thirty-two years old; father of four children; family history was good. His health had always been good as a young man. On a few occasions in his youth he had vaingloriously shown his ability to chew and swallow glass. Thirteen weeks before I saw him he complained of difficulty in swallowing, and six weeks before he found he could not swallow solid food. From the time he first had trouble until the time I saw him he lost between eight and ten pounds. I passed upon him an esophageal bougie, the bulbous end of which was the size of a twenty-five French sound. Obstruction was met about ten inches from the mouth, I judged at the esophageal junction with stomach. I made therapeutic test of a possible syphilitic stricture at the lower end of the esophagus by giving him iodide of potassium in large doses during the next few weeks. His weight declined quite rapidly, and I failed to pass the smallest bougie sometime in December. In January, I made gastrostomy for this man by Witzel's method to save him from starvation. The operation was quite successful. I found constriction of the esophagus dense and firm at its entry into the stomach, with induration and massive infiltration of the cardiac end of the stomach in the neighborhood. Diagnosis of cancer was made. Within a few weeks he picked up rapidly in flesh on gastric feeding through a tube, and for two months bade fare to regain his former health. He then began to complain of pulmonary symptoms with cough, rapid respiration and later expectoration of mucopurulent character, finally becoming foul and hemorrhagic. He died three months and ten days after the gastrostomy of malignant metastasis of the lungs from the primary carcinoma of the esophogeal end of the stomach.

Under the third of the leading indications for surgical treatment of gastric ulcer, that is, under the heading of mechanical interference with motility of the stomach, gastroenterostomy must first be discussed. It is the operation indicated in the majority of cases of ulcer of the stomach. Mayo reports 135 gastrojejunostomies with one death. Acute gastric ulcer may demand it, operation being done for hemorrhage or for perforation, depending upon the site of the ulcer. Chronic ulcer nearly always must be treated this way. In the majority of cases the ulcer is found near the pyloric end of the stomach, and the disturbing consequences of adhesions, induration, infiltration and cicatricial contraction interfere, therefore, with the proper emptying of the cardiac or storage end of the stomach.

Operation of choice is a posterior gastrojejunostomy. Lifting the transverse colon and laying it upward upon the abdomen, the transverse mesocolon is nicked and stretched widely enough to open the lesser cavity of the peritoneum, thus reaching the posterior surface of the stomach. The jejunum is joined to the stomach by a row of seromuscular silk or celluloid sutures. The incision is now made into the stomach and jejunum and is to be an inch and a half to two inches long. Through and through suture of the cut edges is now made so as to appose the edges of the orifices. Then the ends of the original seromuscular suture which are left eight to ten inches long are picked up and carried on to appose seromuscular surfaces around the point of union. As this operation is now done by Moynihan, Mayo and others, no lateral loop anastomosis between the distal and proximal loops of intestine near the site of the gastrojejunostomy is necessary. By drawing the loop of jejunum out straight and attaching it in direct line to the posterior wall of the stomach with capacious opening, at least two inches long as already indicated, the "vicious circle" of the old operative methods is no longer encountered.

On September 15, 1906, I made this operation on Mr. T., a patient of Dr. Woehnert's. Drainage for pyloric obstruction was the operative indication, developing at the end of four years of dyspepsia, the last three months of which were months of practical starvation. Emaciation was pronounced. The pyloric end of the stomach was involved in a fairly large rounded mass of dense adhesions, about the size of a lemon, and was firmly bound to the under surface of the liver and posterior wall of the abdomen. The mass was not nodular in character. Glandular development was not noticeable. The condition seemed to me inflammatory rather than malignant. I decided against a Rodman's operation, and the conditions were not favorable for a pylorectomy. I made a posterior gastrojejunostomy by Moynihan's method. Upon reaching the posterior surface of the stomach, well away from the pylorus, we found a cicatrix about an inch and a half long, due to old ulcer very plainly visible in that situation. The patient did well after the operation. Writing to me a few weeks ago he said he had gained thirty pounds in flesh and was attending to his business without trouble.

Finney's operation or gastroduodenostomy or a pyloroplasty may be done for benign pyloric stenosis.

I did a pyloroplasty for a patient, Miss G——, Dr. Mead referred to me on October 14, 1903. She said she was 25 years old, had been subject to bilious attacks for eight or nine years, lasting two or three days to a week. She had been careless in eating always, being fond of pie and cake. Her weight at her best was 150 pounds. November 20, 1902, she vomited from five till eight o'clock, and it was thought shee had taken some poison. A week later ulcer of the stomach was diagnosticated, when she vomited blood, and all during the winter of 1902 upon eating she vomited. She lost in weight, falling under one hundred pounds. In April, 1903, she had an attack of jaundice, and on consultation with a surgeon, gall-bladder disease was diagnosticated, it being said gallstones were felt. Operation, however, was refused. She gained in flesh after April and had no more vomiting, but she had constant pain under her ribs and in her right side with cramp-like exacerbations at night which were brought on also by eating meat. I found her weighing 110

pounds, tender over the pyloric end of the stomach and over the region of the gall-bladder with moderate rigidity on pressure. I could not make out a tumor. I operated October 29, finding no evidence of gall-bladder disease, but constriction and hardening of the pyloric end of the stomach. On opening the pylorus longitudinally I found a benign stenosis of the pylorus. Making a free incision about two inches in length, I sewed up the incision laterally, making a pyloroplasty. Her digestion improved promptly and her health was soon restored. She has had no trouble since and is now in good health.

Certain cases of gastric ulcer are favorable ones to do resection of the pylorus with anostomosis between duodenum and stomach. The following unusual case of this kind is worthy of report:

On November 13, 1902, Dr. Crosby referred Mrs. Newman to me, a woman fifty-seven years old, who had had twelve children; menopause over at forty-seven. She had long been a dyspeptic with vomiting, belching and rumbling of gas. For some months she had been losing flesh, in all fifty pounds. She suffered severe cramps after taking food.

On examination, a peristaltic wave of large size could be readily seen in the upper abdomen seemingly in the transverse colon. Cramps occurred with this peristalsis. There was a tumor like an enlarged gall-bladder between the navel and the free border of the ribs. Considering age, loss of flesh and tumor, I made a diagnosis of cancer of the hepatic flexure of the colon, and advised against operation so far as possibility of cure was concerned. I agreed to operate, however, because of the pain associated with the obstructed peristaltis already mentioned. It seemed to me an intestinal anastomosis short circuiting the area of bowel involved might give relief. On November 19, 1902, I operated making an incision as for a gall-bladder operation. The mass mentioned was readily reached and proved to be great omentum and colon, which were readily lifted off and separated from the pyloric end of the stomach, which was found thickened and indurated. Considering this condition one of cancer, I made a pylorectomy, cutting away the mass freely into healthy tissue. She made a good recovery. Gastric dilatation and muscle spasm accounted for the violent colicy pain and peristaltic wave observed through the abdominal wall. Microscopic examination in the cancer laboratory by Dr. Gaylord showed the condition to be one of inflammatory induration due to pyloric ulcer of the stomach. She gained in weight and health until May, 1906, when she began to have return of the obstructive condition already mentioned. In the next few weeks the trouble became acute again. Returning to Buffalo for further operation when I was out of town, Dr. Frederick made a gastrojejunostomy from which she recovered. Considering the mass he found involving the pylorus malignant, he made an unfavorable prognosis as to cure. By letter, Dr. Crosby informs me she has regained her normal weight and is now well.

I have not encountered a case of "hour glass" stomach in my experience. It is due to cicatricial contraction following ulcer between the cardiac and pyloric ends of the stomach, making sacculation of the stomach on both sides of the contracture or stricure. Stagnation and decomposition of food occur in each pocket. In future cases of this kind I will do the operation suggested by Mayo, resecting the hour glass area, and bringing together the cut edges. To make the cut edges nearly meet, the line of incision on the pyloric side is made slanting and excess of length still present on the cardiac side is taken up as slack, stitch by stitch, the length of stitch on the cardiac side being sufficiently longer each time to take up the excess in the total length of the suturing. It seems to me the most rational way to effect a cure. It removes the diseased area in which malignancy might otherwise later develop and it avoids the danger of insufficient drainage of the pockets, which gastrogastrostomy or single or double gastroenterostomy often invites.

Lastly, the operation of gastroplication may be briefly discussed. It is not a drainage operation and to me it seems its indication is not found in gastric ulcer or its sequelæ. It would be indicated in atonic dilatation if such a thing exists. I can imagine a condition of excessive gastric dilatation due to pyloric stenosis, in which in addition to gastroenterostomy a gastroplication might be indicated.

1018 Main Street.

